# miR-296 inhibits the metastasis and epithelial-mesenchymal transition of colorectal cancer by targeting S100A4

**DOI:** 10.1186/s12885-017-3121-z

**Published:** 2017-02-16

**Authors:** Zheng He, Lianhua Yu, Shiyi Luo, Mingzhen Li, Junbo Li, Qi Li, Yi Sun, Chengbin Wang

**Affiliations:** 1 Department of Clinical Laboratory, Chinese PLA General Hospital, No. 28 Fuxing Road, Beijing, 100853 China; 2 Department of Laboratory Medicine, Taizhou Municipal Hospital, Taizhou, 318000 China; 3 State Key Laboratory of Physical Chemistry of Solid Surfaces and College of Chemistry and Chemical Engineering, Xiamen University, Xiamen, 361005 China; 4 Beijing Center for Physical and Chemical Analysis, Beijing, 100094 China; 5 Department of Clinical Laboratory, Xiyuan Hospital, China Academy of Chinese Medical Sciences, Beijing, 100091 China

**Keywords:** miR-296, Colorectal cancer, Metastasis, Epithelial-mesenchymal transition, S100A4

## Abstract

**Background:**

Dysregulation of microRNAs (miRNAs) is actively involved in the pathogenesis and tumorigenicity of colorectal cancer (CRC). miR-296 was found to play either oncogenic or tumor suppressive role in human cancers. However, the status of miR-296 and its function in CRC remain unknown.

**Methods:**

The expression of miR-296 was confirmed by qRT-PCR in CRC tissues and cells, and its level was altered by corresponding miRNA vectors. Wound healing and Transwall assays were performed to detect the migration and invasion of CRC cells. The levels of proteins were measured using immunoblotting, immunohistochemistry and immunofluorescence.

**Results:**

Underexpression of miR-296 was disclosed in CRC tissues and cells. Its decreased level was evidently correlated with adverse clinical parameters and poor prognosis of CRC patients. In vitro experiments indicated that miR-296 inhibited CRC cell migration and invasion. Mechanically, miR-296 inhibited the epithelial-mesenchymal transition (EMT) of CRC cells. A negative correlation between miR-296 and S100A4 expression was observed in CRC tissues. Luciferase reporter assays indicated that miR-296 inversely regulated the luciferase activity of 3’-UTR of S100A4. Herein, S100A4 was found to be a downstream molecule of miR-296 in CRC. Furthermore, S100A4 mediated the anti-metastatic effects of miR-296 on EMT, migration and invasion of CRC cells.

**Conclusions:**

miR-296 functions as an anti-metastatic factor mainly by suppressing S100A4 in CRC. It potentially acts as a prognostic predictor and a drug-target for CRC patients.

**Electronic supplementary material:**

The online version of this article (doi:10.1186/s12885-017-3121-z) contains supplementary material, which is available to authorized users.

## Background

Colorectal cancer (CRC), which is the third most common cancer worldwide, is the fourth most common cause of cancer-related deaths [1]. Although remarkable progresses have been made in the diagnosis and treatment of CRC, the survival of CRC patients is still dismal [2]. Systemic metastasis and postoperative recurrence are the main reasons for the unsatisfactory prognosis of CRC patients [3]. However, the molecular mechanisms underlying the metastasis and recurrence of CRC remain largely unknown. Therefore, it is of great significance to understand the molecular mechanisms for the metastasis and recurrence of CRC.

microRNAs (miRNAs) inhibit the expression of target genes by contributing to the degradation or translational inhibition of target mRNAs [4]. They have been found to be actively involved in different cellular processes [5, 6] including cell proliferation, apoptosis, differentiation and movement. Emerging studies show that abnormal expression and dysfunction of miRNAs play important roles in the pathogenesis and tumorigenicity of human malignancies [7–9]. Otherwise, miRNAs have been demonstrated to be hopeful diagnostic biomarkers and drug-targets of CRC [10–12]. Investigating the expression and biological function of miRNAs in CRC will contribute to the discovery of new biomarkers and drug-targets for CRC patients.

Recently, miRNA-296 was found to play important roles in various human cancers including lung cancer [13, 14], glioblastoma [15], bladder cancer [16], and laryngeal carcinoma [16]. Study of lung cancer showed that miR-296 could inhibit the proliferation and enhanced the apoptosis of lung cancer cells [13, 14]. And miR-296 played a suppressive role in glioblastoma by inhibiting glioblastoma cell stemness [15]. However, miR-296 expression contributed to the resistance to radiotherapy and tumor recurrence of laryngeal carcinoma [16], indicating an oncogenic role of miR-296 in laryngeal carcinoma. These indicate that miR-296 plays different roles in different cancer types. Previous study has reported that decrease in blood miR-296 predicts chemotherapy resistance and poor clinical outcome in patients receiving systemic chemotherapy for metastatic colon cancer [17]. In azoxymethane (AOM)-treated rat model, miR-296-5p was downregulated in the uninvolved colonic mucosa (tumor field) of AOM rats [18]. However, the expression and biological role of miR-296 in CRC remain unknown.

Here, we confirmed that miR-296 was underexpressed in CRC specimens and cells. The low level of miR-296 correlated with adverse clinical features of CRC patients and decreased survival rate. Our data showed that miR-296 inhibited the mobility and epithelial-mesenchymal transition (EMT) of cancer cells in CRC. Moreover, S100A4 was identified as a downstream molecule of miR-296 and mediated the biological functions of miR-296 in CRC.

## Methods

### Clinical tissues

Clinical specimens were obtained from 90 histologically diagnosed CRC patients in the Chinese PLA General Hospital. Patients who received immunotherapy, chemotherapy or radiotherapy before surgical treatment were excluded. All specimens were stored in liquid nitrogen or fixed with formalin for further investigation. The demographic features and clinicopathologic date were shown in Table 1.

**Table 1:** Clinicopathological findings and correlation with miR-296 expression in CRC

Features		n = 90	miR-296 expression		*P*
			Low	High	
Age (years)	<65	59	30 (50.85%)	29 (49.15%)	0.824
	≥65	31	15 (48.39%)	16 (51.61%)	
Sex	Male	48	26 (54.17%)	22 (45.83%)	0.398
	Female	42	19 (45.24%)	23 (54.76%)	
Tumor grade	G1 + G2	67	35 (52.24%)	32 (47.76%)	0.468
	G3 + G4	23	10 (43.48%)	13 (56.52%)	
Size (cm)	<5	30	17 (56.67%)	13 (43.33%)	0.371
	≥5	50	28 (56.00%)	32 (44.00%)	
Tumor invasion	T1 + T2	22	5 (22.73%)	17 (77.27%)	0.003^*^
	T3 + T4	68	40 (58.82%)	28 (41.18%)	
Lymph node status	<1	48	16 (33.33%)	32 (66.67%)	0.001^*^
	≥1	42	29 (69.05%)	13 (30.95%)	
Distant metastasis	Absent	69	30 (43.48%)	39 (56.52%)	0.025^*^
	Present	21	15 (71.43%)	6 (28.57%)	
TNM stage	I + II	43	16 (37.21%)	27 (62.79%)	0.020^*^
	III + IV	47	29 (61.70%)	18 (38.30%)	

*CRC* colorectal cancer, *TNM* tumor-node-metastasis. ^*^ Statistically significant

### Cell culture and transfection

Human colorectal cancer cell lines including HCT116, Caco-2, HT29,SW620, and SW480, and human intestinal epithelial cells (HIEC) were obtained from the Institute of Biochemistry and Cell Biology, Chinese Academy of Sciences (Shanghai, China). All cells were cultured in DMEM (HyClone, Logan, UT, USA) along with fetal bovine serum (10%) (FBS; HyClone), penicillin (100 U/ml; Sigma, St-Louis, MO, USA), and streptomycin (100 μg/ml; Sigma). Cell cultures were kept in a incubator containing of 5% CO2 and humidified atmosphere at 37 °C.

miR-296 mimic, miR-296 inhibitor, S100A4 siRNA and the corresponding control vectors were bought from Genecopoeia (Guangzhou, China) and were then transduced into CRC cells with lippofectamine 2000 (Invitrogen, Carlsbad, CA, USA) following manufactures’ protocols. Retroviral vector pMMP-S100A4 was constructed by inserting the corresponding cDNA into pMMP. The retroviruses were packaged and tranfected into CRC cells as previously described [19].

### Quantitative real-time polymerase chain reaction (qRT-PCR)

Total RNA from CRC cells was extracted by miRNeasy Mini Kit (Qiagen, Hilden, Germany) and total RNA from CRC tissues were extracted with Trizol reagent (Ambion, Austin, TX, USA). miR-296 levels in these samples were assayed using TaqMan MicroRNA assays based on the manufacturer’s instructions (Applied Biosystems, Carlsbad, CA). The primers for miR-296 and U6 were obtained from Genecopoeia (Guangzhou, China). U6 was used as the control gene for the relative level of miR-296.

### Luciferase reporter assay

To investigate whether miR-296 could interact with the 3’-UTRs of S1004, wild type (wt) 3’-UTR of S100A4 predicted to interact with miR-296 or the mutant (mt) S100A4 3’-UTR was amplified and cloned into plasmids. Then, the wt 3’-UTR of S100A4 or mt 3’-UTR of S100A4, and miR-296 mimic or miR-296 inhibitor were co-transduced into CRC cells by lippofectamine 2000 (Invitrogen). 48 h after co-transfection, the cells were lysed and assayed using Dual-Luciferase® Reporter Assay Kit (Promega, Madison, WI, USA) based on the manufacturer’s protocols.

### Wound healing assay

CRC cells transfected with corresponding vectors were seeded in 6-well plates to form the single confluent cell layer. The wound were made with 100 μl tips in the confluent cell layer. 0 and 12 h after would scratching, the width of wound was photographed with phase-contrast microscope.

### Transwell migration and invasion assay

The migratory and invasive ability of CRC cells were evaluated with Transwell chambers (BD Biosciences, Franklin Lakes, NJ, USA). 5–10 × 10^4^ CRC cells that were suspended in 100 μl medium without serum were seeded into the upper chamber, and lower chamber was full of 20% FBS to induce CRC cells migrating or invading through the membrane. Matrigel (1:6 dilution; BD Biosciences) was added on the upper chamber for invasion assay. 24 h later, cells with crystal violet (MedChem Express, Shanghai, China) staining that migrated or invaded across the Transwell membrane were counted under optical microscope.

### Western blot

Cell proteins were collected with RIPA lysis buffer (Santa Cruz Biotechnology, Inc., Santa Cruz, CA, USA), and 40ug protein were subjected to 4-20% SDS gel electrophoresis (Sigma) and were then transferred to PVDF membranes (Roche, Indianapolis, IN, USA). Then, 5% milk blocked membranes were incubated with S100A4 (1:1000, Abcam, Cambridge, UK), E-cadherin (1:500, Abcam) or Vimentin (1:1000, Abcam) antibody and subsequently incubated with matched secondary antibodies (Cell signaling, Danvers, MA, USA). Then, signals for each protein expression were detected with the Bio-Rad Gel imaging system (Bio-Rad, Hercules, CA, USA). GAPDH (G8140, US Biological, Swampscott, MA, USA) was used as a loading control.

### Immunofluorescence (IF)

CRC cells were seeded on chamber slides and were fixed with 4% paraformaldehyde for 10 min at room temperature. Then, cells were incubated with antibodies against E-cadherin (1:50, Abcam) or Vimentin (1:100, Abcam) at 4 °C overnight. Then, the slides were incubated with matched secondary antibodies (Invitrogen) at room temperature for 1 h. The nuclear of CRC cells were stained with DAPI (Sigma) at room temperature for 10 min. Fluorescence confocal images were captured using a LSM 5 Pa Laser Scanning Microscope(Zeiss Germany, Oberkochen, Germany).

### Immunohistochemistry (IHC)

Before IHC staining, CRC tissues were fixed with 10% formalin and embedded with paraffin. Then, the embedded tissues were cut into 4 μm thick sections. IHC staining following the standard protocol was performed to evaluate the expression level of S100A4, E-cadherin, and Vimentin in CRC tissues.

### Statistical analysis

All data were collected and showed as Mean ± SEM. Statistical analyses including Pearson chi-squared test, a two-tailed Student’s *t* test, Kaplan-Meier analysis, Log-rand test, Cox regression analysis and Spearman’s correlation analysis were performed with GraphPad Prism 5 software (GraphPad Software, Inc, San Diego, CA, USA) were used in this study to perform statistical analysis. *P* < 0.05 was considered to be statistically different.

## Results

### miR-296 expression is decreased in CRC

**Fig. 1:**
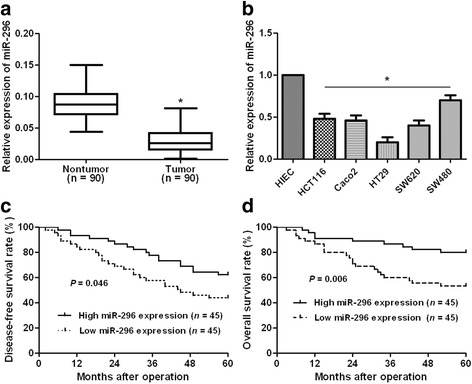
The status and prognostic value of miR-296 expression in CRC. **a** The expression differences of miR-296 between CRC tissues and normal tumor-adjacent tissues. n = 90. **b** The expression differences of miR-296 between 5 different CRC cells lines (HCT116, Caco2, HT29, SW620, SW480) and HIEC cells. *P < 0.05 versus HIEC cells. **c** and **d** Compared with those of high miR-296 level (n = 45), miR-296 low-expressing patients (n = 45) had significantly reduced overall survival and recurrence-free survival

To examine the status of miR-296 in CRC, qRT-PCR was performed on 90 CRC cases. Our data disclosed that CRC tissues showed significant decreased expression levels of miR-296 (*P* < 0.05, Fig. 1a). Next, we compared the expression levels of miR-296 between CRC cells lines and HIEC cells. Compared with HIEC cells, the levels of miR-296 in all CRC cells (HCT116, Caco2, HT29, SW620 and SW480) were significantly reduced (*P* < 0.05, Fig. 1b). These data indicate miR-296 probably plays a suppressive role in CRC.

### Underexpression of miR-296 correlates with adverse clinical parameters and poor prognosis of CRC patients

To clarify the clinical value of miR-296 in CRC, all patients were grouped into miR-296 low and high expression groups according to the median expression of miR-296. As shown in Table 1, CRC patients with low expression of miR-296 had high tumor invasion stage (*P* = 0.003), increased lymph node metastasis (*P* = 0.001) and distant metastasis (*P* = 0.025), and advanced tumor-node-metastasis (TNM) stage (*P* = 0.020). Furthermore, survival analyses indicated that patients with low expression of miR-296 showed significantly reduced 5-year overall and recurrence-free survival (*P* = 0.046 and *P* = 0.006, respectively, Fig. 1c and d). The Cox-regression analysis showed that lymph node status, distant metastasis, TNM stage and miR-296 expression were independent factors for the overall survival and disease free survival of CRC patients (*P* < 0.05, respectively, Table 2). We suggest that miR-296 is a possible prognostic biomarker for CRC patients.

**Table 2:** Multivariate analysis for OS and DFS of 90 CRC patients

Variables	OS			DFS		
	HR	95% CI	*P*	HR	95% CI	*P*
Tumor invasion (T1/T2 *vs* T3/T4)	0.82	(0.33-2.00)	0.658	0.77	(0.31-1.90)	0.567
Lymph node status (<1 *vs* ≥1)	2.35	(1.25-4.41)	0.008^*^	2.43	(1.30-4.59)	0.006^*^
Distant metastasis (Absent *vs* Present)	7.89	(4.58-13.61)	<0.001^*^	9.41	(5.49-16.16)	<0.001^*^
TNM stage (I/II *vs* III/IV)	1.61	(1.01-2.59)	0.049^*^	1.77	(1.10-2.86)	0.019^*^
miR-296 expression (Low *vs* High)	0.44	(0.22-0.86)	0.016^*^	0.43	(0.27-1.02)	0.038^*^

*OS* overall survival, *DFS* disease-free survival, *CRC* colorectal cancer, *TNM* tumor-node-metastasis. ^*^ Statistically significant

### miR-296 inhibits the mobility of CRC cells

**Fig. 2:**
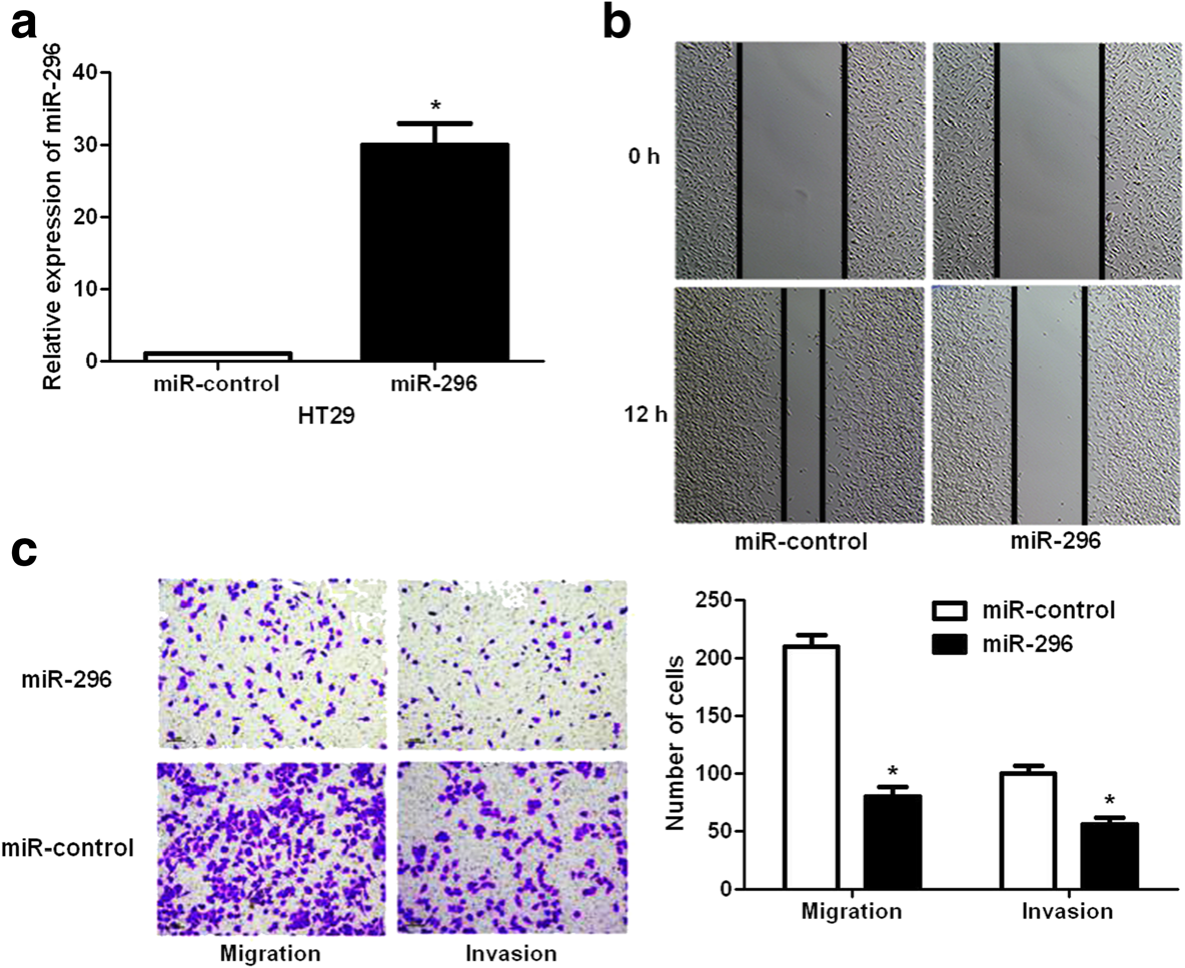
miR-296 overexpression inhibits the mobility of HT29 cells. **a** HT29 cells that were transduced with negative control mimics (miR-control) or miR-296 mimics were confirmed by qRT-PCR. n = 3, *P < 0.05. **b** Wound healing assays indicated that miR-296 overexpression reverses the migration of HT29 cells. **c** Transwell assays confirmed that miR-296 overexpression inhibited HT29 cell migration and invasion. n = 3, *P < 0.05

**Fig. 3:**
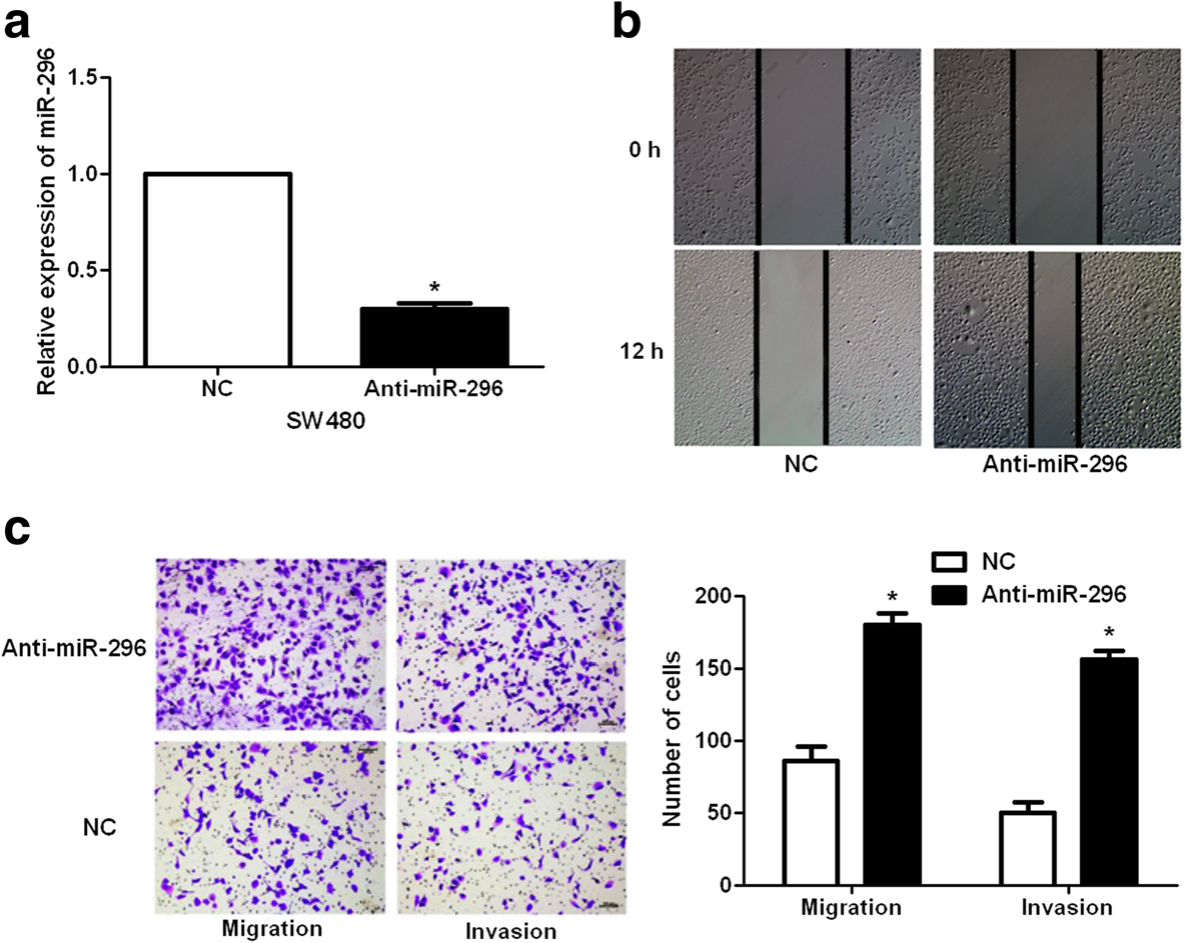
miR-296 knockdown facilitates the metastasis of SW480 cells. **a** SW480 cells that were transduced with negative control inhibitors (NC) or miR-296 inhibitors (anti-miR-296) were confirmed by qRT-PCR. n = 3, *P < 0.05. **b** miR-296 knockdown notably facilitated the migration of SW480 cells. **c** miR-296 knockdown prominently promoted SW480 cell migration and invasion. n = 3, *P < 0.05

Since increased cancer cell mobility is a important reason for the metastasis and recurrence of human cancer [20], we explored whether miR-296 could modulate the migration and invasion of CRC cells. Transfection of miR-296 mimic obviously up-regulated the level of miR-296 in HT29 cells (*P* < 0.05, Fig. 2a). The wound healing assays showed that miR-296 overexpression notably reduced cell migration in HT29 cells (Fig. 2b). And Transwell assays explored that ectopic expression of miR-296 significantly reduced the numbers of migrated and invaded HT29 cells (*P* < 0.05, respectively, Fig. 2c). In turn, miR-296 inhibitor significantly decreased the level of miR-296 in SW480 cells (*P* < 0.05, Fig. 3a). Subsequently, miR-296 silencing notably facilitated SW480 cell migration and invasion (*P* < 0.05, respectively, Fig. 3b and c). Thus, miR-296 exerts a anti-metastatic role in CRC cells.

### miR-296 regulates S100A4 expression and epithelial-mesenchymal transition in CRC cells

**Fig. 4:**
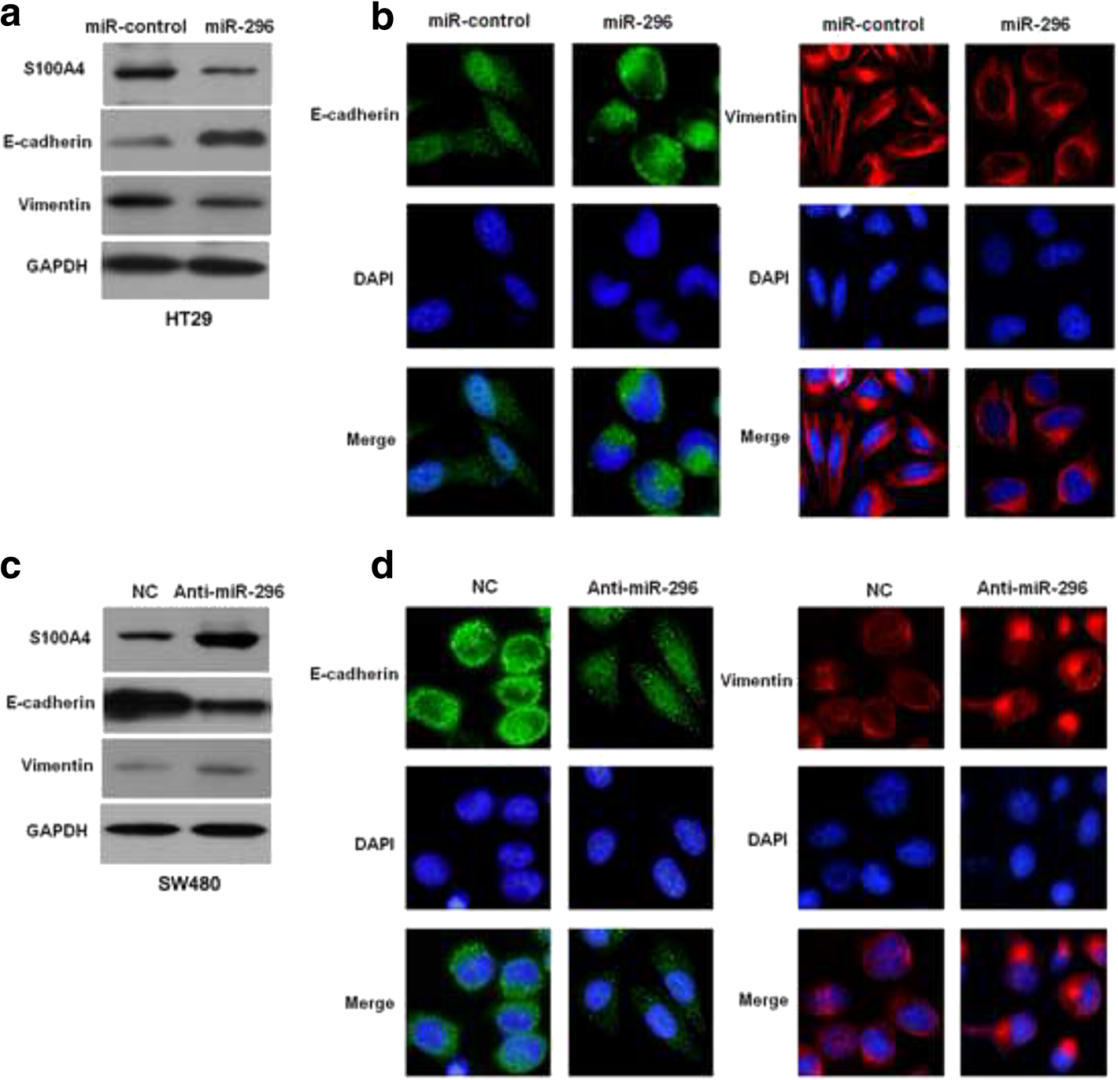
miR-296 inhibits the epithelial-mesenchymal transition of CRC cells. **a** Western blot showed overexpression of miR-296 decreased S100A4 and Vimentin expression, and increased E-cadherin expression in HT29 cells. **b** Immunofluorescence showed miR-296 overexpression increased E-cadherin expression and decreased Vimentin expression in HT29 cells. **c** Western blot showed knockdown of miR-296 increased S100A4 and Vimentin expression, and decreased E-cadherin expression in SW480 cells. **d** Immunofluorescence showed knockdown of miR-296 decreased E-cadherin expression and increased Vimentin expression in SW480 cells

To disclose the underlying molecular mechanisms involved in the role of miR-296 in CRC cells, TargetScanHuman 7.1 (http://www.targetscan.org) was used for discovery of the target molecule of miR-296. EMT has been confirmed to be a critical mechanism for cancer metastasis [21, 22]. S100A4, a marker of EMT phenotype [23] and also a critical regulator of cancer growth and metastasis [24, 25], was recognized as a potential target molecule of miR-296, because the complementary sequence of miR-296 was identified in the 3’-UTR of S100A4 mRNA by TargetScan analysis. Further experiments were performed to confirm the above hypothesis. Western blot showed that miR-296 overexpression in HT29 cells significantly increased the level of epithelial marker E-cadherin, and decreased the expression of mesenchymal markers including Vimentin and S100A4 (Fig. 4a). And the immunostaining accordingly showed that miR-296 overexpression evidently increased E-cadherin expression and decreased Vimentin expresssion (Fig. 4b). On the other hand, miR-296 silencing in SW-480 cells prominently decreased E-cadherin expression, and increased Vimentin and S100A4 expression (Fig. 4c). And immunofluorescence data showed that E-cadherin expression was decreased while Vimentin expression was increased after miR-296 knockdown (Fig. 4d).

**Fig. 5:**
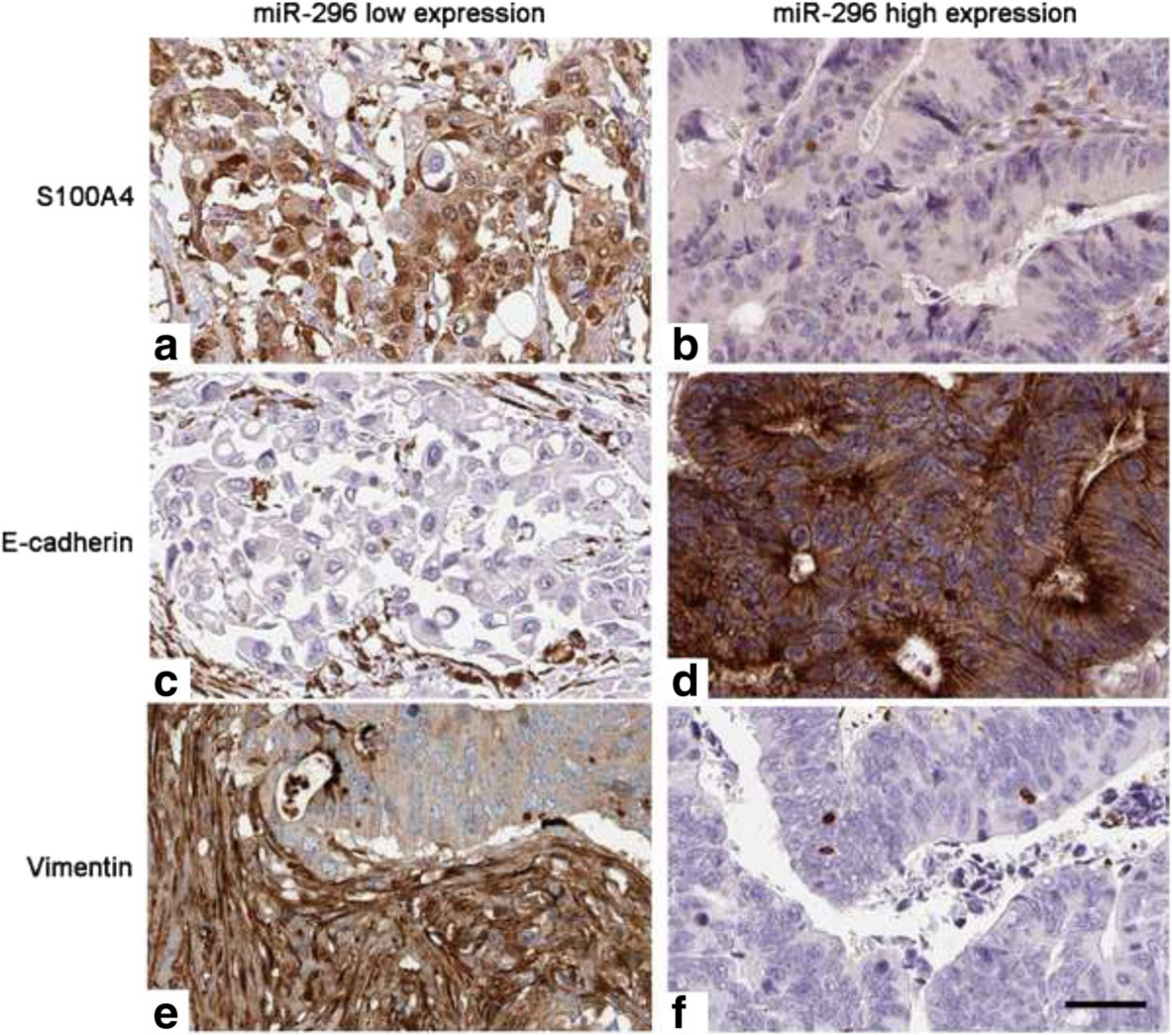
The expression of S100A4, E-cadherin, and Vimentin in CRC tissues. In representative immunohistochemical staining, miR-296 low expressing tumors showed strong staining of (**a**) S100A4 and (**e**) Vimentin, and weak staining of (**c**) E-cadherin. However, miR-296 high expressing tumors showed weak staining of (**b**) S100A4 and (**f**) Vimentin, and strong staining of (**d**) E-cadherin. Scale bar: 50 μm

To further confirm that miR-296 could modulate EMT phenotype of CRC, IHC was performed in CRC tissues for S100A4, E-cadherin and Vimentin. miR-296 low expressing tumors showed strong staining of S100A4 (Fig. 5a) and Vimentin (Fig. 5e), and weak staining of E-cadherin (Fig. 5c). However, miR-296 high expressing tumors showed weak staining of S100A4 (Fig. 5b) and Vimentin (Fig. 5f), and strong staining of E-cadherin (Fig. 5f). Spearman’s correlation analysis indicated that miR-296 was strongly correlated with S100A4 (r = −0.784, *P* = 0.002), E-cadherin (r = 0.531, *P* = 0.013) and Vimentin (r = −0.638, *P* = 0.028) expression in CRC specimens. These data indicate that miR-296 can inhibit the metastasis of CRC cell by modulating EMT phenotype.

### miR-296 post-transcriptionally regulates S100A4 expression

**Fig. 6:**
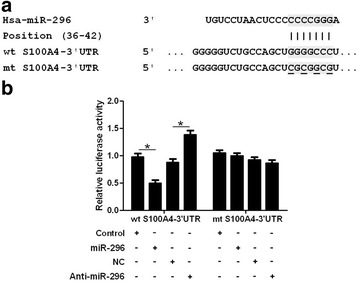
miR-296 binds to the complementary sequence in S100A4 3’-UTR. **a** The binding sites for miR-296 in wild type (wt) and mutant (mt) 3’-UTR of S100A4. **b** Overexpression of miR-296 decreased while inhibition of miR-296 increased the luciferase activity of wt 3’-UTR of S100A4. Alteration of miR-296 had no effect on the luciferase activity of mt 3’-UTR of S100A4. n = 3, *P < 0.05

We further explored whether S100A4 was a downstream target molecule of miR-296. As shown in Fig. 6a, the putative bind sites for miR-296 was presented in the 3’-UTR of S100A4. Then, we performed luciferase reporter assays to investigate whether miR-296 could bind to the putative binding sites in the 3’-UTR of S100A4. Overexpression of miR-296 decreased the luciferase activity of wt 3’-UTR of S100A4 (*P* < 0.05, Fig. 6b) while inhibition of miR-296 increased the luciferase activity of wt 3’-UTR of S100A4 (*P* < 0.05, Fig. 6b). And alteration of miR-296 did not have any influence on the luciferase activity of mt 3’-UTR of S100A4 (Fig. 6b). Therefore, these data indicate miR-296 can regulate the expression of S100A4 by directly interacting with its 3’-UTR in CRC.

### S100A4 mediates the anti-metastatic effects of miR-296 on CRC cells

**Fig. 7:**
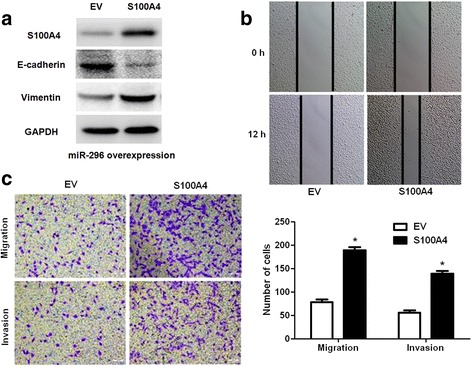
S100A4 restoration reverses the effects of miR-296. **a** miR-296 overexpressing HT29 cells that were infected with empty vector (EV) or S100A4 retroviruses were confirmed by western blotting for S100A4, E-cadherin and Vimentin. **b** S100A4 restoration significantly promoted the migration of miR-296 overexpressing HT29 cells. **c** S100A4 restoration evidently facilitated cell migration and invasion in miR-296 overexpressing HT29 cells. n = 3, *P < 0.05

**Fig. 8:**
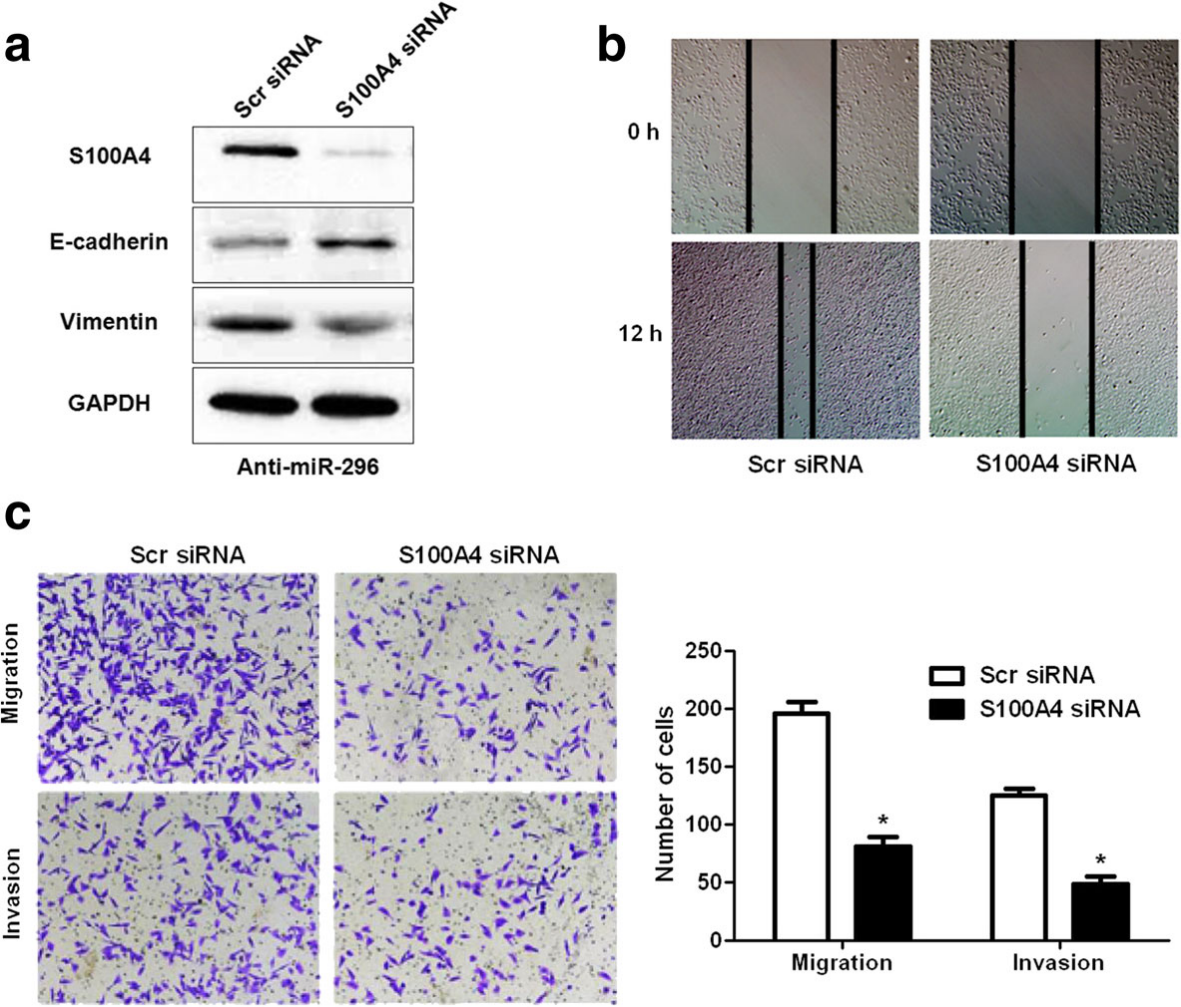
S100A4 knockdown abrogates the effects of miR-296 inhibition on EMT, migration and invasion of CRC cells. **a** miR-296 silenced SW480 cells that were transfected with S100A4 siRNA and scrambled siRNA (scr siRNA) were subjected to western blot. S100A4 siRNA significantly decreased S100A4 in miR-296 silenced SW480 cells, and led to increased E-cadherin expression and decreased Vimentin expression. **b** S100A4 knockdown significantly inhibited the migration of miR-296 silenced SW480 cells. **c** S100A4 knockdown significantly inhibited the migration and invasion of miR-296 silenced SW480 cells. n = 3, *P < 0.05

Since we confirmed S100A4 was a target molecule of miR-296, S100A4 retroviruses were employed to disclose whether S100A4 restoration could abolish the anti-metastatic role of miR-296 in CRC cells. As shown in Fig. 7a, S100A4 retroviruses infection significantly increased the levels of S100A4 and Vimentin while reduced E-cadherin expression in miR-296 overexpressing HT29 cells. Consequently, restoration of S100A4 promoted the metastatic behavior of miR-296 overexpressing HT29 cells with enhanced cell migration and invasion (*P* < 0.05, respectively, Fig. 7b and c). In turn, S100A4 knockdown abolished the effects of miR-296 silencing on EMT, migration and invasion of SW480 cells (*P* < 0.05, respectively, Fig. 8a-c). Since miRNAs usually have multiple targets, further experiments were performed to check whether the cell mobility will be further inhibited or not after over-expression of miR-296 in S100A4 knockdown cells. Our data indicated that miR-296 overexpression slightly reduced the number of migrated and invaded S100A4 knockdown HT29 cells without statistical significance (Additional file 1: Figure S1), suggesting that S100A4 was the major functional target of miR-296. Taken together, these experiments suggest that S100A4 is not only a downstream target but also a mediator of miR-296 in CRC.

## Discussion

Emerging evidences have confirmed that miRNAs are actively involved in the pathogenic process of CRC [26]. And miRNAs have been found to be critical regulators of the metastasis and EMT of CRC cells [27]. According to the important function of miRNAs in CRC, miRNAs have been considered as potential diagnostic biomarkers and drug-targets of CRC [28]. Decreased expression of miR-296 has been found in metastatic colon cancer patients’ blood and AOM-induced CRC rat model [17, 18]. In this study, miR-296 was found to be significantly downregulated in CRC tissues and cells. And decreased expression of miR-296 in CRC tissues conferred malignant clinical features of CRC patients including high tumor invasion stage, lymph node metastasis, distant metastasis, and advanced TNM stage. More importantly, decreased miR-296 was correlated with reduced overall survival and disease-free survival of CRC patients, and was found to be an independent factor for the prognosis of CRC patients. Therefore, miR-296 plays a tumor suppressive role in CRC and could potentially serve as a promising biological tag for the prognosis of patients.

Systemic metastasis is the important reason for the unsatisfactory prognosis of CRC patients [2]. Increased migratory and invasive ability of CRC cells underlies the systemic metastasis of CRC. Therefore, it is of great importance to elucidate the molecular mechanisms for the metastasis of CRC cells. In this study, we found that miR-296 could inhibit migration and invasion of CRC cells in vitro. These data confirm that miR-296 exerts a tumor suppressive role in CRC by inhibiting metastatic behaviors of CRC cells. Moreover, previous studies have showed that EMT is a hallmark of human cancer and is a critical mechanism for cancer metastasis. Therefore, we further investigated whether miR-296 could influence the metastasis of CRC cells by regulating EMT phenotype. Our data showed that overexpression of miR-296 could inhibit EMT of CRC cells while inhibition of miR-296 promoted EMT of CRC cells. Immunohistochemical staining of CRC specimens further confirmed the correlation between the expression of miR-296 and EMT markers. Taken together, these data demonstrate that miR-296 can suppress the metastasis of CRC cells by inhibiting EMT.

S100A4 is a well-known regulator of the growth and metastasis of human cancers [23, 25]. In CRC, S100A4 was found to be aberrantly up-regulated and correlated with the metastatic phenotype of CRC cells [24]. In this study, we found that miR-296 could inhibit the expression of S100A4 in CRC cells. And the expression of S100A4 in CRC tissues was negatively correlated with the expression level of miR-296. Moreover, we found that miR-296 could directly interact with the 3’-UTR of S100A4 using luciferase reporter assay. These data indicate that S100A4 is a direct downstream target of miR-296. Furthermore, we found that restoration of S100A4 reversed the anti-metastatic effects of miR-296 overexpression and S100A4 knockdown abrogate the effects of miR-296 inhibition on EMT and metastasis of CRC cells. Moreover, we disclosed that S100A4 was the major functional target of miR-296 in CRC. These suggest that S100A4 is not only a downstream target but also a mediator of miR-296 in CRC.

All together, our study demonstrates that miR-296 expression is significantly decreased in CRC. The low level of miR-296 correlates with adverse clinical parameters of CRC patients and shortened survival. And miR-296 inhibits the EMT and metastasis of CRC cells. Furthermore, S100A4 is a downstream target of miR-296 in CRC. Altogether, miR-296 exerts its inhibitory effects on CRC metastasis mainly by targeting S100A4.

## Conclusions

Dysfunction of miRNAs has been implicated in the initiation and progression of human cancers. miR-296 was previously found to be a cancer-related miRNAs. Recent study reported that decreased in blood miR-296 predicted chemotherapy resistance and poor clinical outcome in patients receiving systemic chemotherapy for metastatic colon cancer. Yet, the clinical value and biological function of miR-296 remain rarely known in CRC. Here, we presented that miR-296 level in CRC tissues was notably reduced compared to matched non-cancerous specimens. Its decreased level was evidently correlated with adverse clinical parameters including tumor invasion, lymph node metastasis, distant metastasis and advanced TNM stage, and poor prognosis of CRC patients. Accordingly, the levels of miR-296 were obviously down-regulated in CRC cells compared to HIEC cells. Ectopic expression of miR-296 in HT29 cells prominently inhibits the migration and invasion of tumor cells, while miR-296 knockdown increased these behaviors of SW480 cells. Mechanically, miR-296 exerted an anti-metastatic function by suppressing EMT and S100A4 abundance in CRC cells. Herein, S100A4 was found to be a downstream molecule of miR-296 in CRC. A significant correlation between miR-296 and S100A4, E-cadherin and Vimentin was confirmed in CRC specimens. Furthermore, restoration of S100A4 expression could abrogate the anti-metastatic effects of miR-296 on HT29 cells with enhanced cell migration and invasion. S100A4 knockdown inhibited the migration and invasion of miR-296 underexpressing SW480 cells. Altogether, miR-296 potentially act as a prognostic predictor and a drug-target for CRC patients.

## Additional file

Additional file 1: Figure S1. miR-296 overexpression doesn’t notably reduced cell migration and invasion in S100A4 knockdown HT29 cells. S100A4 knockdown HT29 cells that were transfected with negative control mimics (miR-control) and miR-296 mimics, respectively, were subjected to Transwell assays for cell migration and invasion. Quantitative data indicated that miR-296 overexpression slightly reduced cell migration and invasion in S100A4 knockdown HT29 cells. n = 3 repeats with similar results. (TIF 674 kb)

## Abbreviations

CRC: Colorectal cancer
EMT: Epithelial-mesenchymal transition
IF: Immunofluorescence
IHC: Immunohistochemistry
miRNA: microRNA
qRT-PCR: Quantitative real-time polymerase chain reaction
TNM: Tumor-node-metastasis
