# Supplementary figures and images for: miR-296 inhibits the metastasis and epithelial-mesenchymal transition of colorectal cancer by targeting S100A4
Source: BMC Cancer. 2017 Feb 16;17:140. doi: 10.1186/s12885-017-3121-z (PMC5311719; doi:10.1186/s12885-017-3121-z)

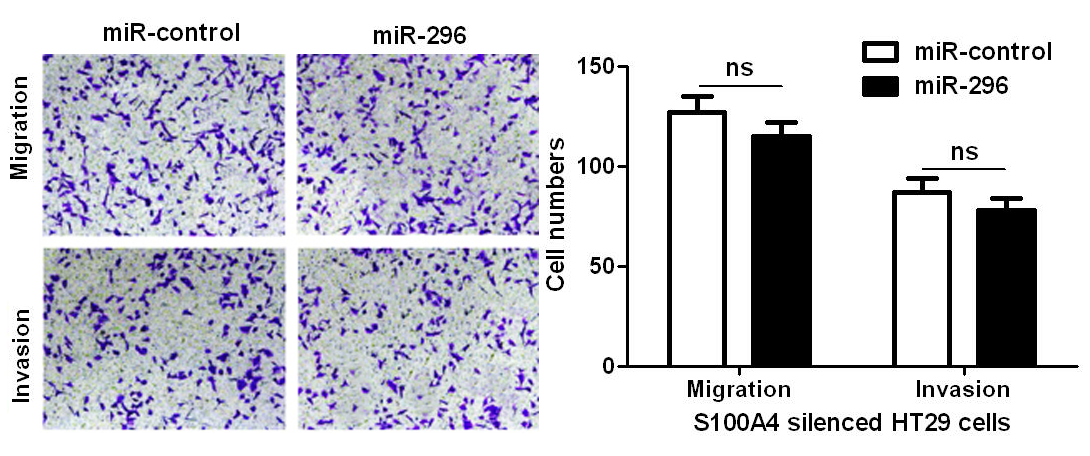

Supplement: Additional file 1: Figure S1. — miR-296 overexpression doesn’t notably reduced cell migration and invasion in S100A4 knockdown HT29 cells. S100A4 knockdown HT29 cells that were transfected with negative control mimics (miR-control) and miR-296 mimics, respectively, were subjected to Transwell assays for cell migration and invasion. Quantitative data indicated that miR-296 overexpression slightly reduced cell migration and invasion in S100A4 knockdown HT29 cells. n = 3 repeats with similar results. (TIF 674 kb) [file 12885_2017_3121_MOESM1_ESM.tif]
